# Safety and efficacy of LA-ERCP procedure following Roux-en-Y gastric bypass: a systematic review and meta-analysis

**DOI:** 10.1007/s00464-023-10276-7

**Published:** 2023-07-21

**Authors:** Baraa Saad, Maya Nasser, Reem H. Matar, Hayato Nakanishi, Danijel Tosovic, Christian A. Than, Stephanie Taha-Mehlitz, Anas Taha

**Affiliations:** 1 St George’s University of London, London, SW17 0RE UK; 2 Division of Gastroenterology and Hepatology, Mayo Clinic, Rochester, USA; 3 School of Biomedical Sciences, The University of Queensland, St Lucia, Brisbane, 4072 Australia; 4 Clarunis, University Centre for Gastrointestinal and Liver Diseases, St. Clara Hospital and University Hospital, 4002 Basel, Switzerland; 5 Department of Biomedical Engineering, Faculty of Medicine, University of Basel, 4123 Allschwil, Switzerland

**Keywords:** RYGB, LA-ERCP, Laparoscopically assisted transgastric ERCP, Gastric bypass, Meta-analysis

## Abstract

**Introduction:**

Rapid weight loss following Roux-en-Y gastric bypass surgery (RYGB) translates to an increased need for endoscopic retrograde cholangiopancreatography (ERCP) intervention. Laparoscopically Assisted Transgastric ERCP (LA-ERCP) has emerged to address the issue of accessing the excluded stomach. This study aims to evaluate the safety and efficacy of LA-ERCP procedure following RYGB.

**Methods:**

The Cochrane, EMBASE, SCOPUS, MEDLINE, Daily and Epub databases were searched from inception to May 2022 using the PRISMA guidelines. Eligible studies reported participants older than 18 years who underwent the LA-ERCP procedure, following RYGB, and outcomes of patients.

**Results:**

27 unique studies met the inclusion criteria with 1283 patients undergoing 1303 LA-ERCP procedures. 81.9% of the patients were female and the mean age was 52.18 ± 13.38 years. The rate of concurrent cholecystectomy was 33.6%. 90.9% of procedures were undertaken for a biliary indication. The mean time between RYGB and LA-ERCP was 89.19 months. The most common intervention performed during the LA-ERCP was a sphincterotomy (94.3%). Mean total operative time was 130.48 min. Mean hospital length of stay was 2.697 days. Technical success was 95.3%, while clinical success was 93.8%. 294 complications were recorded with a 20.6% complication rate. The most frequent complications encountered were pancreatitis (6.8%), infection (6.1%), bleeding (3.4%), and perforation (2.5%). Rate of conversion to open laparotomy was 7%.

**Conclusion:**

This meta-analysis presents preliminary evidence to suggest the safety and efficacy of LA-ERCP procedure following RYGB. Further investigations are warranted to evaluate the long-term efficacy of this procedure using studies with long-term patient follow-up.

**Supplementary Information:**

The online version contains supplementary material available at 10.1007/s00464-023-10276-7.

Obesity continues to be one of the most prominent contemporary medical problems with an increase in bariatric surgery as the most effective treatment for morbid cases [1, 2]. One of the most common bariatric surgeries is Roux-en-Y gastric bypass (RYGB) with more than 150,000 yearly procedures performed worldwide [2].

RYGB alters the normal gastrointestinal anatomy as to exclude most of the stomach, duodenum, and proximal jejunum. This leads to rapid weight loss over the period of 12–18 months, predisposing to complications such as cholelithiasis, choledocholithiasis and gallstone pancreatitis [3–5]. This translates to an increased need for investigation and/or treatment with endoscopic retrograde cholangiopancreatography (ERCP) in RYGB patients [6]. However, the altered anatomy of RYGB patients renders conventional access to the pancreaticobiliary tract more difficult, leading to the rise of alternative ERCP approaches such as rotational and single or double-balloon enteroscope [3, 7]. These approaches are hindered by some major limitations such as the inability to reach the papilla or to cannulate the desired ducts, so novel techniques were implemented [7–10].

Laparoscopic-assisted ERCP (LA-ERCP), the focus of our study, was first described in 2002 [13] and continues to be widely used [14]. It is a single-stage laparoscopic technique that includes a trocar being placed in the remaining stomach followed by insertion of the conventional duodenoscope through the trocar to reach the ampulla of Vater [15]. LA-ERCP is distinguished by its high technical success rate of reaching the major papilla as well as a high clinical success rates, determined by the completion of intended treatment [14, 16, 17]. However, regarding complication and adverse event rates, some studies report low rates [15, 18, 19], whereas other studies report high ones [20–22]. These complications include bleeding, perforation, pancreatitis, and wound infection. These conflicting results raises uncertainties on whether the benefits of LA-ERCP truly outweigh its risks.

Thus, the aim of this systematic review and meta-analysis is to evaluate the efficacy and safety of LA-ERCP in function of its success and complication rates. Types of intervention, total operative time and hospital stay were also valuated. To our knowledge, this is the largest and most comprehensive systematic review and meta-analysis of the aforementioned outcomes.

## Methods

### Search strategy and data sources

A comprehensive search of several databases from inception to May 6, 2022, was conducted and limited to English language only. The databases included Ovid MEDLINE(R) and Epub Ahead of Print, In-Process & Other Non-Indexed Citations, Daily, Ovid EMBASE, Ovid Cochrane Central Register of Controlled Trials, Ovid Cochrane Database of Systematic Reviews, and Scopus. The search strategy was designed and conducted by a medical reference librarian. Controlled vocabulary supplemented with keywords was used to search for studies describing Laparoscopic-assisted ERCP (LA-ERCP) and RYGB.

### Eligibility criteria and quality assessment

Eligible studies must have met all the following inclusion criteria: (1) participants must be older than 18 years who underwent gastric bypass; (2) participants underwent LA-ERCP procedure, (3) Reported primary outcomes of technical or clinical success of procedure or complications/adverse events following the procedure. Technical success of the LA-ERCP procedure was defined as either access to the excluded stomach or the successful cannulation of the intended duct. Clinical success of the EDGE procedure was defined as successful performance of EUS or ERCP. The methodological quality of each study was independently evaluated by two authors (BS and MS) using the ROBINS – I tool [23].

### Statistical analysis

Means of continuous variables and rates of binary variables were pooled using the random-effects model, generic inverse variance method of DerSimonian and Laird [24]. Proportions underwent logit transformation prior to meta-analysis. The heterogeneity of effect size estimates across the studies was quantified using the *Q* statistic and the *I*^2^ index (P < 0.10 was considered significant). A value of *I*^2^ of 0–25% indicates minimal heterogeneity, 26–50% moderate heterogeneity, and 51–100% substantial heterogeneity. Data analysis was performed using Open Meta analyst software (CEBM, Brown University, Providence, Rhode Island, USA). If mean and standard deviation (SD) were unavailable, the median was converted to mean using the formulas from the Cochrane Handbook for Systematic Reviews of Interventions [25].

## Results

### Study selection and characteristics

The initial search yielded 606 potentially relevant articles from which 27 unique studies involving 1283 patients met eligibility criteria [15, 16, 18–22, 26–45]. The details of the study selection process and PRISMA flow diagram are depicted in Supplementary Fig. 1.

### Risk of bias

Results of the quality assessment of all included studies are shown in Supplementary Fig. 2. Overall risk of bias was low in 75% of included studies, while 25% of included studies had a moderate risk of bias.

### Baseline and clinical characteristics

**Fig. 1:**
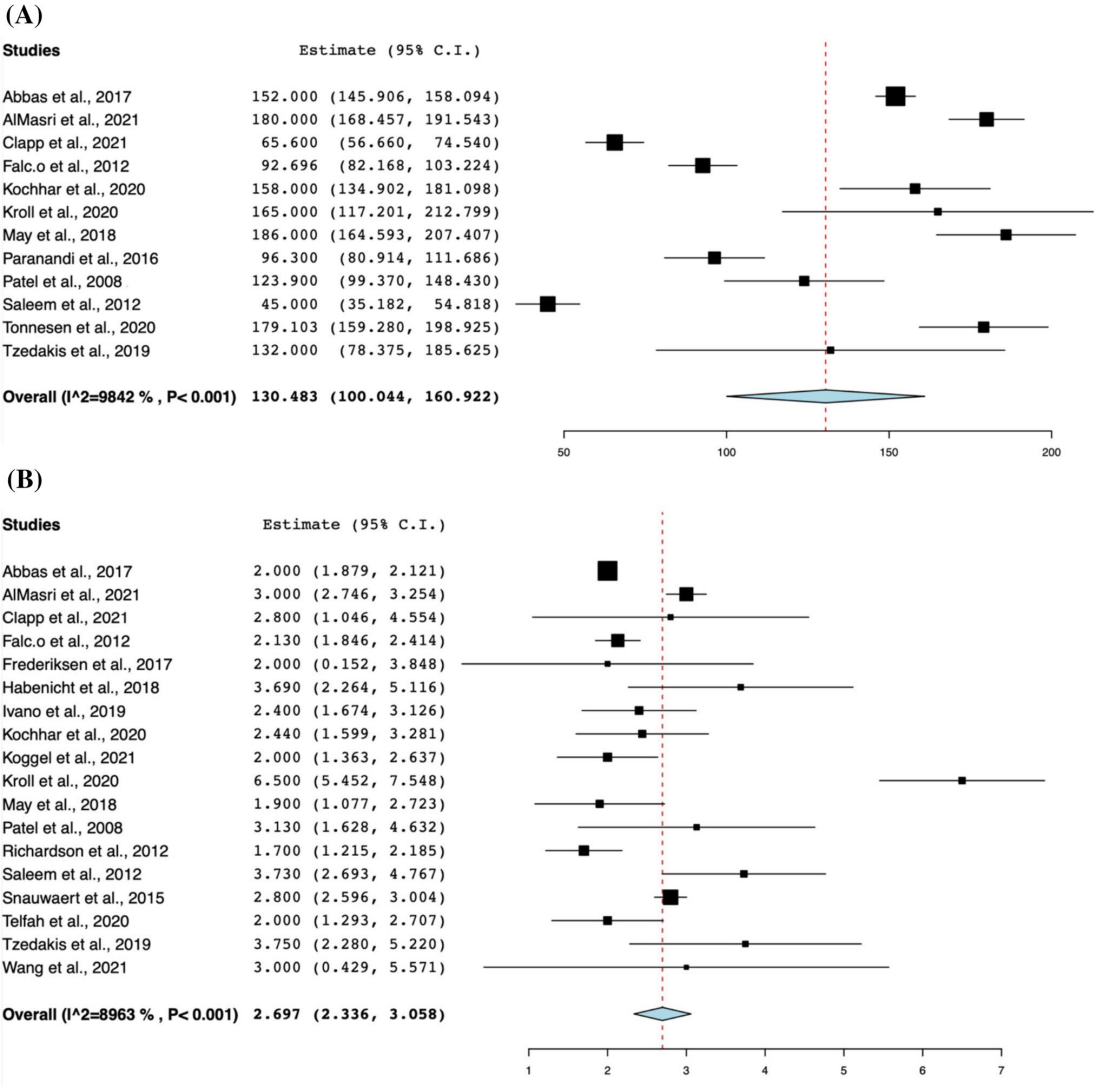
Forest plots of total operative time and length of hospital stay of the LA-ERCP procedure

The baseline characteristics of the included studies are comprehensively described in Table 1. 1283 included patients underwent a total of 1303 LA-ERCP procedures. 1051 patients (81.9%) were female. The mean age of the participants was 52.18 ± 13.38 years. 56.8% of patients (n = 686) had a cholecystectomy prior to LA-ERCP (95% CI 0.474, 0.657; *I*^2^ = 78.85%) [16, 18, 20–22, 26, 28, 30–32, 34, 36–39, 41–44]. 33.6% had a concurrent cholecystectomy with LA-ERCP (n = 326)_(95% CI 0.251, 0.432; *I*^2^ = 81.17%) [15, 16, 19–22, 26–28, 30, 32, 34, 36–39, 41–44]. Of 1247 LA-ERCP procedures, 1149 procedures were undertaken for a biliary indication (90.9%, 95% CI 0.867, 0.938; *I*^2^ = 47.16%) while 80 procedures were undertaken for a pancreatic indication (8.6%, 95% CI 0.058, 0.124; *I*^2^ = 42%), while the rest of the procedures were undertaken for other indications not stated in the papers included. The clinical characteristics of the patients undergoing the LA-ERCP procedure are shown in Table 2. The mean time between RYGB and LA-ERCP was 89.19 months (95% CI 61.03, 117.35; *I*^2^ = 99.68%) [21, 22, 26, 28, 30, 31, 36–38, 44, 45]. The most common intervention performed during the LA-ERCP was a sphincterotomy, being performed 94.3% of the time (95% CI 0.926, 0.956; *I*^2^ = 0%), followed by stone/sludge/cast extraction at 65.9% (95% CI 0.537, 0.764; *I*^2^ = 82.99%), followed by biliary/pancreatic stent placement at 9.7% (95% CI 0.049, 0.184; *I*^2^ = 66.38%), followed by ampulla/papilla/stricture dilation at 5.1% (95% CI 0.02, 0.122; *I*^2^ = 61.4%), and lastly by biliary/pancreatic stent extraction at 5.0% (95% CI 0.036, 0.068; *I*^2^ = 0%). Figure 1 shows the forest plot of total operative time and length of hospital stay of the LA-ERCP procedure. The mean total operative time was 130.48 min (95% CI 100.04, 160.92; *I*^2^ = 98.42%) [16, 18, 20, 26, 28, 33–35, 37, 38, 43, 44]. In addition, the mean hospital length of stay was 2.697 days (95% CI 2.336, 3.058; *I*^2^ = 89.63%) [16, 18–22, 26, 28, 30, 31, 33–35, 38, 41–44].

**Table 1:** Baseline characteristics of included studies and patients

Study	Author	No. of participants	No. of procedures	Gender (female)	Mean age ± SD	Cholecystectomy status		Indication for procedure	
						Previous	Concurrent	Biliary	Pancreatic
Abbas et al.,	USA, Brazil, Canada	579	579	488	51 ± 13.33	423	114	518	45
AlMasri et al., 2021	USA	131	131	106	60 ± 12.59	66	62	128	NR
Bowman et al., 2016	USA	11	11	8	48.8 ± 13.7	NR	3	6	4
Ceppa et al., 2006	USA	5	5	NR	NR	NR	2	5	0
Clapp et al., 2021	USA	12	12	10	44.8 ± 10.6	12	NR	11	1
Falcão et al., 2012	Brazil	23	23	19	35.3 ± 6.7	13	10	23	0
Frederiksen et al., 2017	Denmark	29	31	25	46 ± 10	15	12	31	0
Grimes et al., 2014	USA	38	38	36	47.8	NR	NR	NR	NR
Habenicht et al., 2018	USA	16	17	NR	55.8 ± 9.5	11	5	17	0
Ivano et al., 2019	Brazil	7	7	4	43.5 ± 14.6	3	NR	7	0
Kedia et al., 2018	USA	43	44	36	55 ± 11.75	16	23	36	7
Kochhar et al., 2020	USA	18	18	12	60.78 ± 12.67	NR	NR	19	2
Koggel et al., 2021	Netherlands	86	100	70	53.5 ± 11.25	46	36	100	0
Kroll et al., 2020	Switzerland	14	14	11	45.5 ± 11	6	8	14	0
May et al., 2018	USA	51	51	45	55.4 ± 10.9	NR	NR	51	0
Mohammad et al., 2020	USA	32	32	26	54 ± 13	17	6	30	2
Paranandi et al., 2016	UK	7	7	7	50.28 ± 14.4	5	0	7	0
Patel et al., 2008	USA	8	8	7	44 ± 10.7	7	1	6	2
Richardson et al., 2012	USA	11	11	9	54.27 ± 12.55	NR	1	8	3
Roberts et al., 2008	USA	5	5	5	44.6 ± 9.5	5	0	5	0
Saleem et al., 2012	USA	15	15	12	50.9 ± 12.6	8	3	14	1
Schreiner et al., 2012	USA	24	24	19	52	NR	NR	23	1
Snauwaert et al., 2015	Belgium	23	23	18	54 ± 13.25	10	13	19	4
Telfah et al., 2020	UK	12	12	9	64 ± 9.75	10	1	12	0
Tonnesen et al., 2020	Norway	37	39	27	48.8 ± 13	10	25	38	1
Tzedakis et al., 2019	France	4	4	4	41.25 ± 10.66	3	1	3	1
Wang et al., 2021	USA	42	42	38	50.6 ± 15.9	NR	NR	37	5

*NR* not reported

**Table 2:** Clinical characteristics of LA-ERCP procedure

Study	No. of procedures	Mean time between RYGB and LAERCP (months) ± SD	Mean total operative time (min) ± SD	Mean hospital stay (days) ± SD	Interventions performed during LAERCP				
					Sphincterotomy	Extraction of stone/sludge/cast	Dilation (ampulla/papilla/stricture)	Stent placement (biliary/pancreatic)	Stent extraction (biliary/pancreatic
Abbas et al., 2017	579	NR	152 ± 74.81	2 ± 1.48	550	253	147	126	30
AlMasri et al., 2021	131	81.6 ± 63.1	180 ± 67.4	3 ± 1.48	128	102	0	7	0
Bowman et al., 2016	11	NR	NR	NR	9	5	0	2	0
Ceppa et al., 2006	5	NR	NR	NR	4	1	0	0	0
Clapp et al., 2021	12	56.4 ± NR	65.6 ± 15.8	2.8 ± 3.1	12	NR	NR	NR	NR
Falcão et al., 2012	23	16.34 ± 5.22	92.7 ± 25.76	2.13 ± 0.69	23	17	0	0	0
Frederiksen et al., 2017	31	36 ± 21	NR	2 ± 5.25	NR	31	NR	NR	NR
Grimes et al., 2014	38	NR	264.8 ± NR	4.2 ± NR	NR	NR	NR	NR	NR
Habenicht et al., 2018	17	82.8 ± 3.25	NR	3.69 ± 3	15	15	0	1	1
Ivano et al., 2019	7	516 ± 54.7	NR	2.4 ± 0.98	7	7	0	7	0
Kedia et al., 2018	44	NR	184 ± 84.5	2.65 ± NR	43	NR	NR	NR	NR
Kochhar et al., 2020	18	NR	158 ± 50	2.44 ± 1.82	NR	NR	NR	NR	NR
Koggel et al., 2021	100	27 ± 44.25	80 ± 43.75	2 ± 3.25	92	54	NR	NR	NR
Kroll et al., 2020	14	NR	165 ± 91.25	6.5 ± 2	14	14	NR	NR	NR
May et al., 2018	51	NR	186 ± 78	1.9 ± 3	NR	NR	NR	NR	NR
Mohammad et al., 2020	32	64.2 ± 88.2	NR	NR	32	31	0	1	0
Paranandi et al., 2016	7	43.14 ± 37.89	96.3 ± 20.8	NR	6	5	0	0	1
Patel et al., 2008	8	38.26 ± 16.07	123.9 ± 35.4	3.13 ± 2.17	8	2	0	0	0
Richardson et al., 2012	11	NR	NR	1.7 ± 0.82	11	6	0	0	0
Roberts et al., 2008	5	NR	NR	NR	5	1	0	0	0
Saleem et al., 2012	15	NR	45 ± 19.4	3.73 ± 2.05	15	3	0	1	0
Schreiner et al., 2012	24	NR	172	1.67 ± NR	NR	NR	NR	NR	NR
Snauwaert et al., 2015	23	NR	NR	2.8 ± 0.5	23	17	NR	NR	NR
Telfah et al., 2020	12	NR	NR	2 ± 1.25	10	9	1	1	0
Tonnesen et al., 2020	39	NR	179.1 ± 63.2	NR	NR	NR	NR	NR	NR
Tzedakis et al., 2019	4	31.75 ± 31.7	132 ± 54.7	3.75 ± 1.5	NR	4	NR	NR	NR
Wang et al., 2021	42	100.8 ± 62.4	NR	3 ± 8.5	NR	NR	NR	NR	NR

*NR* not reported

### Outcomes of LA-ERCP procedure

**Fig. 2:**
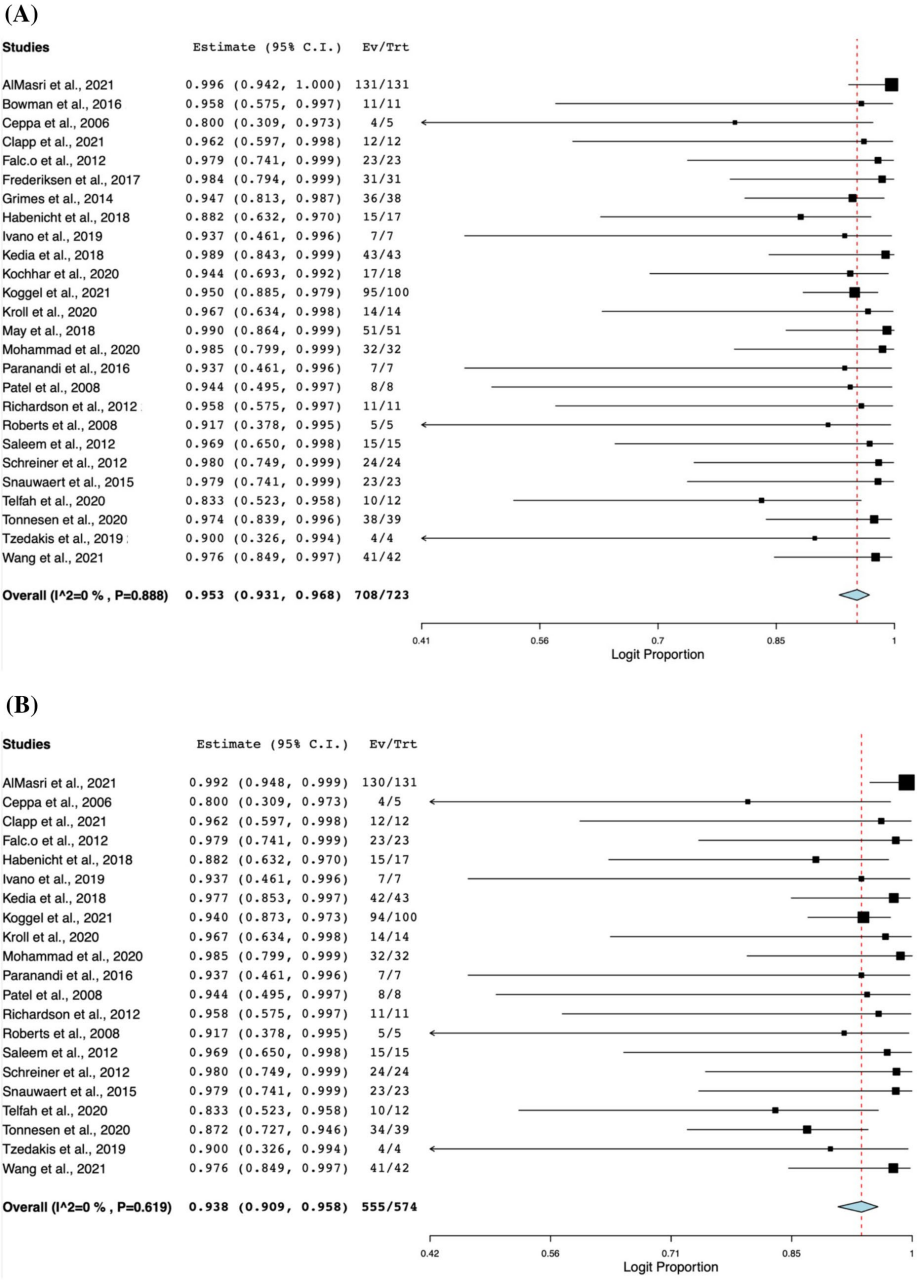
Forest plots of success rates of the LA-ERCP procedure

**Fig. 3:**
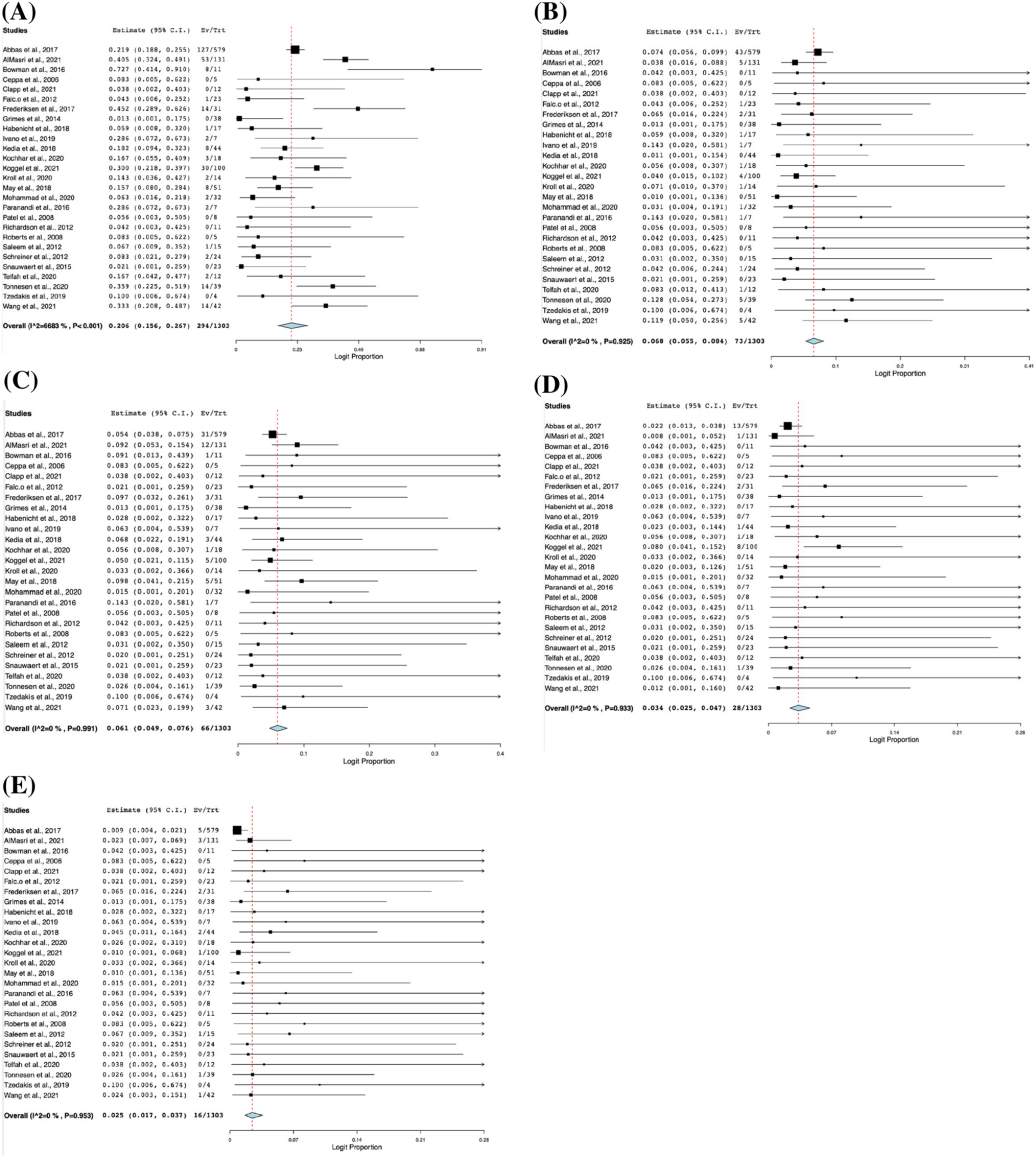
Forest plots of complication rates of the LA-ERCP procedure

The outcomes of the LA-ERCP procedure are depicted in Table 3. The pooled technical success of LA-ERCP was 95.3% (95% CI 0.931, 0.968; *I*^2^ = 0%) [15, 16, 18, 19, 21, 22, 26–45], while the clinical success of the LA-ERCP procedure was 93.8% (95% CI 0.909, 0.958; *I*^2^ = 0%) [15, 16, 18, 19, 22, 26, 28, 30–32, 34, 36–45]. Figure 2 demonstrates the forest plots of success rates of the LA-ERCP procedure. Out of 1303 procedures, 294 complications were recorded (20.6%, 95% CI 0.156, 0.267; *I*^2^ = 66.83%). Moreover, the four most frequent complications encountered were pancreatitis with 73 incidents at 6.8% (95% CI 0.055, 0.084; *I*^2^ = 0%), followed by infection with 66 incidents at 6.1% (95% CI 0.049, 0.076; *I*^2^ = 0%), followed by bleeding (Intraoperative ERCP and Laparoscopic bleeding and post-op bleeding) with 28 incidents at 3.4% (95% CI 0.025, 0.047; *I*^2^ = 0%), and perforation with 16 incidents at 2.5% (95% CI 0.017, 0.037; *I*^2^ = 0%). Figure 3 shows the forest plots of complication rates of the LA-ERCP procedure. Other complications with lower rates include cholangitis, gastric site leak, ileus, cardiovascular and respiratory adverse events, wound dehiscence, bowel obstruction, nerve entrapment and abdominal pain. Table 4 shows the pooled rates of other complications of the LA-ERCP procedure. Out of 275 complications, 226 were classified as minor or moderate or Clavien–Dindo Grade I or II (73.7%, 95% CI 0.604, 0.837; *I*^2^ = 60.27%), while 49/275 were classified as severe, or life-threatening or Clavien-Dindo Grade III or IV (26.3%, 95% CI 0.163, 0.396; *I*^2^ = 60.27%) [16, 20–22, 26, 30, 32, 34, 35, 37, 40, 43, 45]. The pooled rate of conversion to open laparotomy was 7% (95% CI 0.056, 0.088; *I*^2^ = 0%) with 67/1213 procedures undergoing this conversion [15, 16, 18–22, 26–32, 34–42, 44, 45].

**Table 3:** Outcomes of LA-ERCP procedure

Study	N	Technical success *N* (%)	Clinical success *N* (%)	Conversion to open procedure *N* (%)	Number of complications (n)	Types of complications				
						Pancreatitis	Infection	Bleeding	Perforation	Other
Abbas et al., 2017	579	NR (98%)	NR (98%)	29 (5.3%)	127	43	31	13	5	35
AlMasri et al., 2021	131	131 (100%)	130 (99.2%)	14 (10.68%)	53	5	12	1	3	32
Bowman et al., 2016	11	11 (100%)	NR	1 (9%)	8	0	1	0	0	7
Ceppa et al., 2006	5	4 (80%)	4 (80%)	1 (20%)	0	0	0	0	0	0
Clapp et al., 2021	12	12 (100%)	12 (100%)	0 (0%)	0	0	0	0	0	0
Falcão et al., 2012	23	23 (100%)	23 (100%)	0 (0%)	1	1	0	0	0	0
Frederiksen et al., 2017	31	31 (100%)	NR	2 (6.45%)	14	2	3	2	2	5
Grimes et al., 2014	38	36 (95%)	NR	1 (2.63%)	0	0	0	0	0	0
Habenicht et al., 2018	17	15 (94%)	15 (94%)	1 (5.88%)	1	1	0	0	0	0
Ivano et al., 2019	7	7 (100%)	7 (100%)	0 (0%)	2	1	0	0	0	1
Kedia et al., 2018	44	43 (100%)	42 (97.7%)	4 (9.3%)	8	0	3	1	2	2
Kochhar et al., 2020	18	17 (94%)	NR	NR	3	1	1	1	0	0
Koggel et al., 2021	100	95 (95%)	94 (94%)	0 (0%)	30	4	5	8	1	12
Kroll et al., 2020	14	14 (100%)	14 (100%)	1 (7.14%)	2	1	0	0	0	1
May et al., 2018	51	51 (100%)	NR	1 (1.96%)	8	0	5	1	0	2
Mohammad et al., 2020	32	32 (100%)	32 (100%)	0 (0%)	2	1	0	0	0	1
Paranandi et al., 2016	7	7 (100%)	7 (100%)	0 (0%)	2	1	1	0	0	0
Patel et al., 2008	8	8 (100%)	8 (100%)	2 (25%)	0	0	0	0	0	0
Richardson et al., 2012	11	11 (100%)	11 (100%)	0 (0%)	0	0	0	0	0	0
Roberts et al., 2008	5	5 (100%)	5 (100%)	0 (0%)	0	0	0	0	0	0
Saleem et al., 2012	15	15 (100%)	15 (100%)	2 (13.33%)	1	0	0	0	1	0
Schreiner et al., 2012	24	24 (100%)	24 (100%)	3 (12.5%)	2	1	0	0	0	1
Snauwaert et al., 2015	23	23 (100%)	23 (100%)	2 (8.7%)	0	0	0	0	0	0
Telfah et al., 2020	12	10 (83.3%)	10 (83.3%)	2 (16.67%)	2	1	0	0	0	1
Tonnesen et al., 2020	39	38 (97.4%)	34 (87.18%)	NR	14	5	1	1	1	6
Tzedakis et al., 2019	4	4 (100%)	4 (100%)	0 (0%)	0	0	0	0	0	0
Wang et al., 2021	42	41 (98%)	41 (98%)	1 (2.38%)	14	5	3	0	1	5

*NR* not reported

**Table 4:** Summarized complication rates

Complication	Estimate (%)	95% CI	*I*^2^ (%)
Pancreatitis	6.8	0.055–0.084	0
Infection	6.1	0.049–0.076	0
Bleeding	3.4	0.025–0.047	0
Perforation	2.5	0.017–0.037	0
Cardio-respiratory events	2.5	0.017–0.036	0
Abdominal pain	4.1	0.027–0.061	0
Cholangitis	2.1	0.013–0.032	0
Gastric site leak	1.9	0.012–0.029	0
Bowel obstruction	2.3	0.014–0.038	0
Wound dehiscence	2.5	0.015–0.041	0
Ileus	2.4	0.015–0.041	0
Nerve entrapment	2.3	0.014–0.037	0

## Discussion

The search for an optimal procedure allowing for ERCP to be performed in patients with RYGB anatomy is ongoing. Thus, the primary aim of this systematic review and meta-analysis was to investigate the efficacy and safety of the laparoscopic-assisted ERCP (LA-ERCP) following RYGB. To our knowledge, this is the most comprehensive systematic review and meta-analysis to evaluate the efficacy and safety of LA-ERCP as a function of its success and complication rates. Types of intervention, total operative time and hospital stay were also comprehensively evaluated.

A total of 27 studies including 1283 patients and 1303 LA-ERCP procedures were included. Our study demonstrated that the LA-ERCP procedure is feasible, efficient, and has high technical and clinical success rates.

The pooled overall technical success in our study was considerably high and similar to the rate of success of ERCP in normal GI anatomy [46]. This translates to a high rate of successful cannulation of the desired duct in LA-ERCP and thus correlates to the high clinical success rate.

It is important to view LAERCP outcomes in the context of results obtained from other techniques currently used in RYGB patient management as this can influence the choice of procedure and healthcare provided to patients. EUS-guided transgastric ERCP (EDGE) is a novel technique introduced by Kedia et al. in 2014 [11]. Another recently popular approach is laparoscopic trans-cystic common bile duct exploration (LTCBDE) [47].

In patients with RYGB anatomy, balloon enteroscopy ERCP (BE-ERCP) had a lower technical success rate of 71.4%, while EDGE and LTCBDE had a similar rate of 95.5% and 90,9% respectively [12, 48]. However, LTCBDE can be limited by the size of the bile duct stone more commonly being used with stones < 4 mm and less frequently in stones > 8 mm [49]. Da Ponte-Neto postulates that the high success rates of LA-ERCP are due to firstly, the use of standard duodenoscopes allowing for better tangential visualization of the papilla and use of other appropriate ERCP accessories, and secondly due to the use of an elevator allowing for better access to the papilla, both of which are not used in balloon enteroscopy-based techniques [14].

However, despite the high success rates, the pooled rate of overall complications was shown to be relatively high. Similar results are found in a previous meta-analysis with an overall complication rate of 18% and a similar distribution of complications [12].

LA-ERCP post ERCP pancreatitis (PEP) rates are comparable to conventional ERCP [46]. Risk factors for increased rates of PEP are prolonged or difficult cannulation and mechanical trauma to the pancreatic sphincter causing subsequent edema [46]. The use of wire guided biliary cannulation as well as the use of pancreatic stents can reduce the risk of PEP, however placing stents can be difficult and carries its own risks [46].

Our overall rate of complications is higher than the rate of complications in ERCPs performed in normal GI anatomy [46]. The complication rate is comparable with EDGE and LTCDBE but is higher than BE-ERCP (9.9%) [12, 14, 49]. However, procedures like EDGE have further limitations such as the need for a 2-stage procedure as well as the possibility of permanently forming a gastro-gastric fistula that affects the integrity of the RYGB, and a relatively high complication rate including formation of gastro-gastric fistulas [3, 12]. Importantly, the majority of complications in our study (73.7%) were classified as mild or moderate or Clavien-Dindo I or II. This carries important clinical significance as the incidence of severe or life-threatening complications is relatively low. The higher complication rate in LA-ERCP can possibly be explained due to the laparoscopic procedure itself carrying certain risks not present in endoscopic procedures, such as wound and tube site infections, laparoscopic-related bleeding, and laparoscopy-related perforations [29]. Additionally, Abbas et al. demonstrated that procedures converted to open laparotomy had an increased risk of complications [20]. In our study, the rate of conversion of LA-ERCP procedures to open laparotomies was comparable with current literature [14]. The most common predictors to conversion to open surgery is the presence of adhesions in the setting of multiple prior abdominal operations and a large decrease in BMI between RYGB and LA-ERCP with odds ratios of 10.4 and 1.1 respectively [26]. Furthermore, if a patient had a concurrent cholecystectomy during LA-ERCP, this could certainly influence the adverse event rate, which our analyses showed to be higher. Moreover, factors like the placing of an indwelling G-tube and the use of periprocedural antibiotics could have a impact on the rate of complications [20]. This warrants further large, two-arm controlled research assessing the factors contributing to the complication rate, with clear distinctions between ERCP related and laparoscopic related adverse events. On the other hand, certain studies have shown a reduced rate of complications and higher rate of papilla access by performing a rendezvous procedure using trans-cystic guided cannulation [50]. Although the potential benefits of this novel technique are promising, there have been limited studies in the literature to support its effectiveness. A well-designed, large prospective, two-arm study or randomized control trial is necessary to further our understanding and determine the clinical benefits for patient outcomes.

In our study, the pooled total operative time for LA-ERCP was similar to that of LTCDBE but longer than that of BE-ERCP, and EDGE (55–80 min) [11, 48, 51, 52]. The shorter procedure duration of endoscopic methods achieved in BE-ERCP come at the expense of a lower success rates [14]. The heterogeneity for our pooled operative time is high (98.42%) and this could present an explanation for the discrepancy between our findings and other ERCP modalities. Some studies report median operative time as low as 45 min, and others as high as 180 min. This suggests that the operative time may depend on factors external to the procedure itself such as the expertise of the surgeons, equipment used, patient characteristics, and coordination between the surgeons and the endoscopists. In addition, longer procedure times may be explained by concurrent cholecystectomies, which cannot be performed with different modalities like EDGE and BE-ERCP. This has the advantage of reducing the total number of procedures that a patient has to undergo, however, in certain institutions, the ERCP and the cholecystectomy are conducted by different disciplines which may add logistical challenges, further increasing the procedure time [51]. Additionally, LA-ERCP allows for the diagnosis and concomitant management of adhesions (reported in 20%) and internal hernias [16, 17, 20].

Hospital stay length similarly follows the same trend, being slightly higher than both EDGE and BE-ERCP, with a reported mean hospital stay of respectively 0.8 days and 1.67 days [11, 40]. This is because EDGE is performed in the endoscopy suite as an outpatient procedure [32]. Although one less day spent in the hospital on average has a fiscal and resource benefit, the benefit of LA-ERCP is it allows for concomitant management of conditions stated above while simultaneously having relatively short and a similar length of stay to EDGE.

Previous studies comparing LA-ERCP and EDGE have been published [12], however, the existing evidence is not sufficient to make a clear determination of their relative effectiveness. A randomized control trial is needed to have a direct comparison and establish a definitive conclusion.

Our systematic review and meta-analysis has some important limitations of note most of which are inherent to any meta-analysis. Firstly, our analysis included retrospective studies, and this contributes to selection bias. Secondly, our study was unable to analyze and validate the long-term outcomes of LA-ERCP due to the lack of data in included studies. As such, it is necessary to follow-up and maintain contact with these patients to evaluate this procedure in the long-term. Additionally, the definition of technical and clinical success rates varied in each study with some studies defining technical success as reaching the papilla, while other cannulating the papilla. Thus, it is important to adopt a unified definition as well as standardized reporting methods to allow for less heterogeneity. Finally, this procedure is relatively novel procedure and requires expertise from both the surgeon and the endoscopist. This study does not assess the learning curve for LA-ERCP and does not account for the skill of the surgeon and/or endoscopist, thus possibly introducing heterogeneity and bias to the results. Nevertheless, this study is the largest and most comprehensive available in literature for the LA-ERCP procedure. More studies are warranted to better evaluate the clinical performance of LA-ERCP procedure, especially with respect to its adverse events and the factors that influence them. We also believe that a large randomized controlled trial comparing LA-ERCP, and EDGE is warranted and should be the next step in order to reduce the limitations stated above.

In summary, this meta-analysis presents preliminary evidence evaluating the safety and efficacy of the LA-ERCP procedure in RYGB patients. Despite limited data in this meta-analysis, there appears to be a high technical and clinical success rate. Moreover, there appears to be a promising trend suggesting an acceptable complication rate, length of hospital stay, and efficient operative time. As such, based on the aforementioned results, further studies are required to elucidate the safety and efficacy of the LA-ERCP procedure in a larger number of patients for a longer follow-up period.

## Supplementary Information

Below is the link to the electronic supplementary material.

Supplementary file1 (TIF 25122 KB)

Supplementary file2 (TIFF 5103 KB)

## Data Availability

With the publication, the data set used for this meta-analysis will be shared upon request from the study authors.
