# Supplementary figures and images for: Safety and efficacy of LA-ERCP procedure following Roux-en-Y gastric bypass: a systematic review and meta-analysis
Source: Surg Endosc. 2023 Jul 21;37(9):6682–94. doi: 10.1007/s00464-023-10276-7 (PMC10462525; doi:10.1007/s00464-023-10276-7)

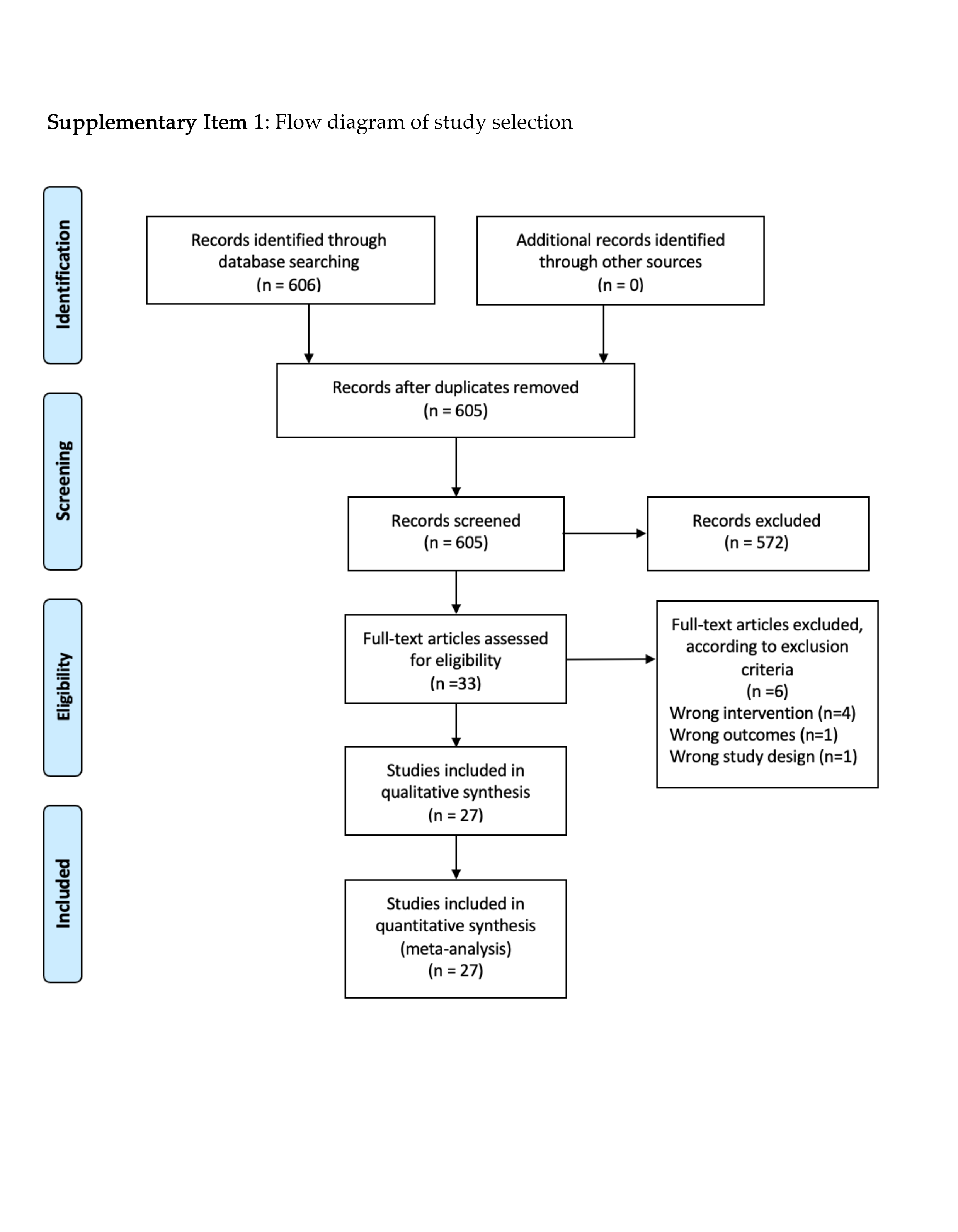

Supplement: Supplementary file 1 — Supplementary file1 (TIF 25122 KB) [file 464_2023_10276_MOESM1_ESM.tif]

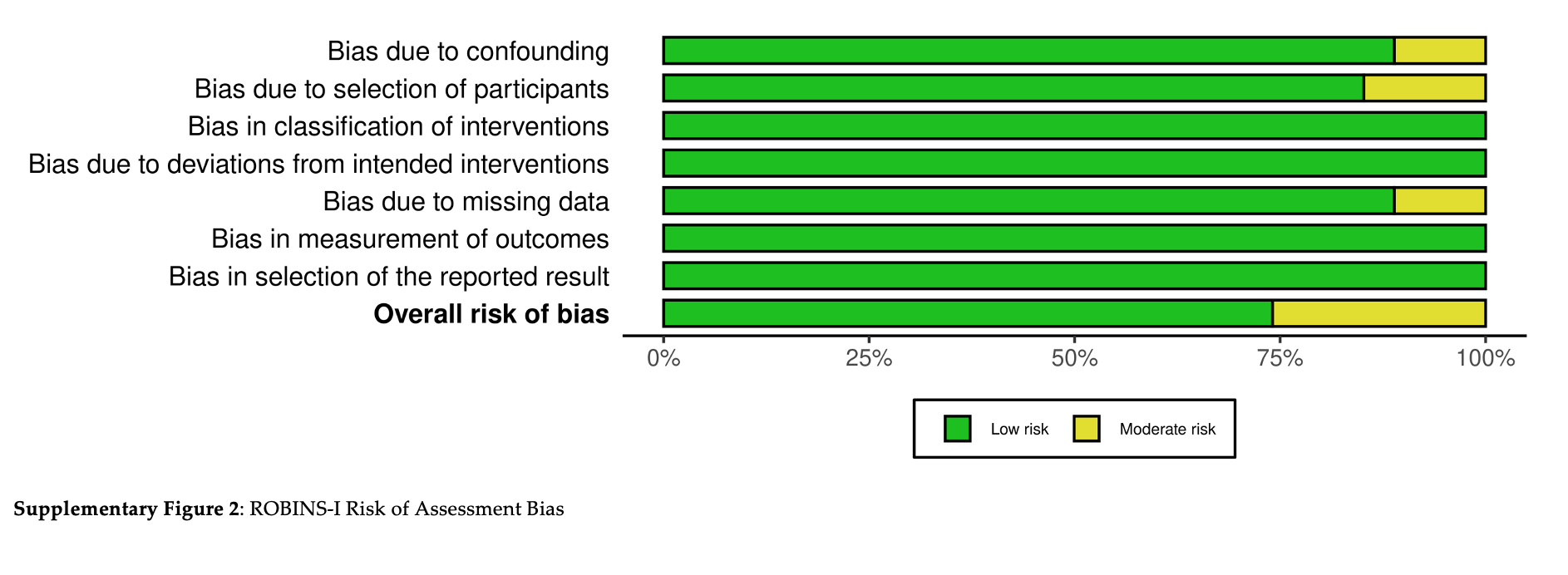

Supplement: Supplementary file 2 — Supplementary file2 (TIFF 5103 KB) [file 464_2023_10276_MOESM2_ESM.tiff]
